# Conversion of no/low value waste frying oils into biodiesel and polyhydroxyalkanoates

**DOI:** 10.1038/s41598-019-50278-x

**Published:** 2019-09-24

**Authors:** Marco Vastano, Iolanda Corrado, Giovanni Sannia, Daniel K. Y. Solaiman, Cinzia Pezzella

**Affiliations:** 1 Dipartimento di Scienze Chimiche, Università Federico II, VIa Cinthia, Napoli, 48126 Italy; 2 Eastern Regional Research Center, Agricultural Research Service, U. S. Department of Agriculture, 600 East Mermaid Lane, Wyndmoor, PA 19038 USA; 3 Dipartimento di Agraria, Università Federico II, Via Università, 100, Portici (Na), Italy

**Keywords:** Environmental biotechnology, Biopolymers

## Abstract

A sustainable bioprocess was developed for the valorization of a no/low value substrate, *i*.*e*. waste frying oils (WFOs) with high content of free fatty acids (FFAs), otherwise unsuitable for biodiesel production. The bioprocess was verified using both recombinant (*Escherichia coli*) and native (*Pseudomonas resinovorans*) polyhydroxyalkanoates (PHAs) producing cell factories. Microbial fermentation of WFOs provided a 2-fold advantage: *i*) the reduction of FFAs content resulting into an upgrading of the “exhausted waste oils” and *ii*) the production of a bio-based microbial polymer. Proper strain designing and process optimization allowed to achieve up to 1.5 g L^−1^ of medium chain length, mcl-PHAs, together with an efficient conversion (80% yield) of the treated WFO into biodiesel.

## Introduction

Growing interest in sustainable production of goods and energy is pushing the development of new biorefinery model. Biodiesel production from waste frying oils (WFOs) is one of the most representative case study in this field. The terms “vegetable exhaust oil (VEOs)” and “waste frying oils (WFOs)” refer to vegetable oils that have been used in food production and are no longer viable for human use. Waste oils arise from many different sources, including domestic, commercial and industrial ones and represent a problematic waste stream which requires to be properly managed. It is disastrous if dumped onto the soil or if discarded into any water body, since its eco-toxic proprieties could contaminate the soil and damage plants^1^.

Storically, this waste raw material has been recycled in several processes including: (*i*) animal feeding, (*ii*) energy production, (*iii*) oleochemistry and (*iv*) biodiesel production.

As a reaction to the BSE166 scandals in the early 2000s, since 31 October 2004 waste cooking oil from catering premises can no longer be used as an ingredient in animal feed. According to the Animal By-Products Regulation EC 1774/2002 (ABPR), in the EU only WFOs from food manufacturing, and fresh or unused cooking oil, are allowed to be used in animal feed. The oleochemical industry relies on animal fats and WFO for the production of a variety of products ranging from consumer products like shampoo and candles, to plastics and building materials. According to APAG, the European association of the oleochemical industry, the relation between WFO and animal fats used in the industry is 1:9 (i.e. for every 10 tonnes of raw materials, 1 tonne is WFO and 9 tonnes are animal fat). Currently, about the 90% of collected WFOs is destined to biodiesel production^2^.

The growing interest towards this feedstock has led to the establishment of a real market for WFOs and the competition for feedstock supplying is rising. WFO quality can fluctuates depending on the collection source, products that are fried, the frequency of replacing WFO with fresh cooking oil and the vegetable oil used for cooking.

Biodiesel is a mixture of fatty acid acyl esters usually produced from lipid substrates (vegetable oils and animal fats) by transesterification. Currently, most of the commercial biodiesel is produced from vegetable oils by an alkali-catalyzed process. Several aspects including the type of catalyst, alcohol/vegetable oil molar ratio, temperature, purity of the reactants (mainly water content) and FFAs content have an influence on the course of the transesterification. In particular, the last two parameters play a key-role in the success of the reaction having a negative influence when they exceed in the feedstocks^3^. Water helps the formation of FFAs by hydrolyzing triacylglycerols (TAGs) and esters. As a result, FFAs in the presence of basic homogeneous catalysts generate soap, which creates serious technical problems when separating the product and ultimately hindering the catalytic activity^3^. To minimize these inconveniences, refined vegetable oils must be used for the process; otherwise, an additional acid-catalyzed pretreatment step is needed to reduce FFAs and water concentrations under an optimum threshold limit, *i*.*e*., FFAs <5% and water <0.5%. The idea of replacing fresh oils with used vegetable ones in biodiesel production process offers a triple-facet solution: economic, environmental and waste management^4^. The use of WFOs for biodiesel offers a genuinely sustainable outlet for a problematic waste product. The potential of using waste cooking oil as feedstock has been already validated for industrial and especially for homemade biodiesel production. Sodhi and his team have optimized the transesterification process by varying different parameters (methanol:oil ratio, type of catalyst, catalyst concentration and reaction temperature) in order to identify the condition that could give the highest yield (i.e. 93.8% of conversion) of WFOs characterized by a % FFA <0.5^5^. The most favorable condition has resulted in 60 °C, using 1% of catalyst concentration and methanol:oil ratio 5:1. The Open University of Sri Lanka has also explored the same theme and even tried to fabricate a Small and Medium-sized Enterprises (SME) level plant for the production of biodiesel from WFOs. Selected wastes have been subjected to a high-impact pretreatment (60 °C, H_2_SO_4_) since their content of FFAs was too high (>2% FFAs)^6^. Among the possible routes for WFOs exploitation, alternatives to biodiesel production, an emerging area is represented by its utilization as raw material for microbial fermentation. Several studies have already proposed the microbial conversion of fatty acids rich media in renewable fuels and chemicals^7–9^, suggesting that fatty acids could become a sustainable feedstock for industrial production^10,11^. Attention has been payed to the development of WFOs based fermentation for the production of an attractive class of biopolymers: polyhydroxyalkanoates (PHAs).

PHAs are microbial polyesters produced, as carbon and energy storage materials, by several bacteria under unbalanced nutrition conditions. Depending on the chain length, PHAs could be thermoplastic polyesters, elastomers or even sticky resins composed of several R-hydroxyalkanoic acids^12^. Due to their biodegradability and biocompatibility, PHAs represent green alternatives to conventional petroleum-based plastic, finding a range of applications from food packaging to biomedical sectors. In view of process sustainability, the use of waste materials as carbon sources represents a valuable alternative to reduce PHAs production costs. High production levels (20–40% of cell dry weight) of polymers with mixed and variable compositions (from C8 to C16), have been achieved, from *Pseudomonas species* fed on WFO as related C-sources^8,13,14^.

Aim of this work is the set-up of a microbial bioprocess aimed at: (*i*) improving the quality of the supplied WFOs by reducing its FFA content; (*ii*) supporting PHAs production by converting undesired free fatty acids into a biodegradable biopolymer. The feasibility of the designed process has been verified using both recombinant (*E*. *coli*) and native (*P*. *resinovorans*) PHAs producing cell factories.

The process has been validated in terms of biopolymer yield as well as of the effective conversion of the resulting WFOs into biodiesel.

## Materials and Methods

### Strains

*LipoA* and *LipoB* had been described^15^. *P*. *resinovorans* NRL B-2649 was obtained from NCAUR-ARS-USDA (Microbial Culture Collection, Peoria, IL, USA). To prepare the *lip*^*−*^ gene knock-out *P*. *resinovorans* strain, an EZ-Tn5 <KAN-2> transposon insertion reaction was performed with recombinant plasmid pETB1-lip as previously described^16^. To map the site of the transposon insertion, a chromosomal DNA fragment with the transposon-interrupted *lip* gene was amplified by PCR, using Taq polymerase and primers of TransFwd and TransRvs. The larger dimensions of the *lip*^*−*^ amplified fragment than the *wt* one suggested the success of the knock-out, which was further confirmed by DNA sequencing.

### Culture media

The compositions of LB and MM media were described previously^17^. The composition of SB medium is as follows (for 1 L): 100 mL M9 salt [10x]; 0.1 mL of CaCl_2_ [1 mol L^−1^]; 5 mL of Glucose [40%]; 2 mL of MgSO_4_ [1 mol L^−1^]. Composition of M9 salt [10x] for 1 L [pH 7.4] is as follows: 60 g Na_2_HPO_4_; 30 g KH_2_PO_4_; 5 g NaCl; 10 g NH_4_Cl. Composition of Medium E is as follows (for 1 L): (NH_4_)_2_HPO_4_, 1.1 g; K_2_HPO_4_, 5.8 g; KH_2_PO_4_, 3.7 g. The pH was adjusted to 7.0 and the medium was autoclaved^16^. 10 mL of a sterile 100 mmol L^−1^ MgSO_4_ solution and 1 mL of a microelement solution were added. The microelement solution contained the following (per liter of 1 mol L^−1^ HCl): FeSO_4_·7H_2_0, 2.78 g; MnCl_4_·H_2_O, 1.98 g; CoSO_4_·7H_2_O, 2.81 g; CaCl_2_·2H_2_O, 1.67 g; CuCl_2_·2H_2_O, 0.17 g; ZnSO_4_·7H_2_O, 0.29 g. Where applicable, glycerol collected from transesterification reaction of fermented oil was added to final concentration of 0.2 or 0.8%.

### WFOs

The *E*. *coli* experiments were carried out with two kinds of WFOs: household waste oils (WFO-A) and industrial (food services facilities) waste oils (WFO-B). Samples WFO-A0 and WFO-B0 represent the waste as they were at collection time. With the aim to obtain wastes with characteristics closer to those of low value used oils^18^ both WFO-0 were subjected to different stressing processes. WFO-A1 and WFO-B1 underwent a thermal treatment to catalyse the oxidation process which occurs during long cooking time; while the samples WFO-A2 and WFO-B2 represent the naturally aged oil after 5 months. In this way four derived substrates were obtained (Table 1).

**Table 1:** Properties of different WFOs utilized in this work.

ID	Sample	% FFA	Density [kg m^−3^]
A0	WFO-A analysed soon after collection	<1%	863
B0	WFO-B analysed soon after collection	1%	895
A1	WFO-A thermally abused (300 °C, 16 hr)	7%	859
A2	WFO-A naturally aged (8 months)	1%	894
B1	WFO-B thermally abused (300 °C, 16 hr)	3%	905
B2	WFO-B naturally aged (8 months)	5%	910

For *P*. *resinovorans* experiments WFO used as an additional carbon source was provided by FargecoS.r.l (V. Scafatella – 80021 Afragola (NA) – Italia), which specializes in the recovery of vegetable exhaust oils from restaurants, pubs, markets and any other place that produces exhaust oils during its business activities. The oils were stored for 10 months in uncontrolled conditions. The free fatty acids (FFAs) content of the WFOs was estimated by standard titration with KOH. The sample was solubilised in 10 ml of isopropyl alcohol, and phenolphthalein (1%) was used as pH indicator. To calculate FFA% from a titration, the following formula was used:

$$FFA \% =(v-b)\times N\times {28.2}/w$$

*v*, is the volume in ml of titration solution

*b*, is the volume in ml of the blank

*N*, is the normality of the titration solution;

*w*, is the weight of the sample of oil in grams; it was used 1 ml of oil, which typically weighs 0.92 g

*28*.*2*, is the molecular weight of oleic acid divided by ten.

### Microbial bioprocess

Recombinant *E*. *coli* cells from solid culture were inoculated in 20 ml LB medium in a 100 mL shaken flask. This preculture was grown overnight at 37 °C on a rotary shaker (200 rpm), then a volume of suspension sufficient to reach a final optical density (OD_600_) of 0.1 was used to inoculate 250 mL shaken flasks containing 50 mL of each tested growth medium (MM + 12% WFO, SB + 12% WFO). IPTG (at 0.5 mmol L^−1^) induction was performed at a defined time (5 h from the inoculum). Flasks were incubated at 37 °C on a rotary shaker at 250 rpm for 72 h. In all conditions, effective induction of protein expression was verified by performing SDS-PAGE analyses according to standard methods. Cellular pellets were recovered at different growth times (24 h, 48 h, 72 h) and processed as described below.

*P*. *resinovorans* cells were grown in Medium E + 12% of WFO (v/v) at 30 °C, with rotary shaking (250 rpm). Culture broth was prepared in 125 mL volumes in 250 mL flasks. The flasks were inoculated with a 1% inoculum from an overnight culture in LB. Kanamycin (Km, 50 µg mL^−1^) was added only for the growth of *lip*^−^ strain in LB media^16^.

Cellular pellets at different growth times were recovered through centrifugation of the liquid culture at 7,200 RCF. After rinsing twice with an aqueous-isopropanol solution (1:1), followed by a 30-min centrifugation, the precipitate is collected, oven-dried at 60 °C overnight to remove any isopropanol residue, cooled at −20 °C and lyophilized to dryness. Weights of the lyophilized cells constituted the cell dry weights. The supernatant was harvested to manually recover the oil suspended on the surface. A subsequent centrifugation of the recovered oil was performed at 9,300 RCF rpm to remove any liquid culture’s residue. Then samples were stored at 4 °C.

### Oil transesterification

10 mL of oils were placed in a beaker equipped with a magnetic stirrer and a thermometer. Under agitation, the oil was heated up to a desired temperature (60 °C) on a heating plate. The operation was carried out according to a protocol described by Leung and coworkers^19^: 10 min at 190 RCF followed by 10 min at 60 RCF. The products of reaction were allowed to settle overnight producing two distinct liquid phases: crude ester phase at the top and glycerol phase at the bottom. Reactions were performed at a molar ratio of 6:1 (methanol: oil) and with 1% NaOH (wt. %) as catalyst. The crude ester phase, separated from the bottom glycerol phase, was analysed by TLC on silica. A mixture of toluene–chloroform (7:3, v/v) was utilized as the mobile phase. Samples of biodiesel, acylglycerol and free fatty acids (oleic acid, OA) were normalized to 50 mg mL^−1^ in isopropanol. Two µL were then applied to the origin of a 10 × 10 cm chromatoplate. A solution of phosphomolybdic acid (10% in Ethanol) was used for TLC staining.

### Determination of Biodiesel conversion yield

After the transesterification, 50 µL of the glycerol-free reaction, were dissolved in CDCl_3_ (internal standard, for ^1^H: CHCl_3_ at d 7.26 ppm) and analysed by ^1^H NMR on Bruker DRX-400 (^1^H NMR: 400 MHz)^20,21^.

Transesterification yield is calculated directly from the area (A) of the selected signals:

$$Y \% ={100}({2}\times A{1}/{3}\times A{2})$$

where A1 and A2 are the areas of the methoxy (δ 3.6) and the methylene protons (δ 2.3), respectively^20,21^. If necessary, samples were extracted with chloroform to remove any soap/glycerol residual.

### Determination of PHA yield

PHA content and composition were determined by gas chromatography after methanolysis as previously described^15,17^. % PHAs was expressed as the ratio between produced PHAs (mg) and cdw (mg) of lyophilized cell material. For PHA extracted from *E*. *coli*, the composition was also confirmed by ^1^H-NMR analysis of purified products^15^.

For *P*. *resinovorans wt* and *lip*^*−*^ strains, PHA’s content was determined gravimetrically and reported as percentage ratio of mg PHA/mg cdw (% PHA). The biopolymer was extracted by vigorously shaking dry cell pellets (100 mg), mixed with chloroform (30 mL) in a shaking flask overnight at room temperature, with constant stirring. After removing the cell debris by filtration through Whatman no. 1 filter paper, the clear chloroform extract was placed in a fume hood for solvent evaporation. The polymer was then re-dissolved in 5 mL of chloroform and precipitated using 10 volumes of cold methanol as a precipitating solvent.

## Results and Discussion

### Exploitation of recombinant PHA producers in the designed bioprocess

The idea behind this study is that microbial fermentation of WFOs by properly designed strains could give rise to an integrated bioprocess that allows the production of two products: (*i*) PHA biopolymers, and (*ii*) a treated oil, with low FFA content, ready for conversion into biodiesel. The *E*. *coli* recombinant systems *LipoA* and *LipoB*^15,17^ were tested for the validation of the proposed bioprocess. These systems, engineered with the *B*. *cereus* PHA biosynthetic operon in a lipase-free background, have been found able to convert fatty acids into scl-mcl copolymers^15^. The performances of *LipoA* and *LipoB* were evaluated on MM medium supplemented with mimetic WFOs (mWFOs) prepared by doping corn oil with a standard FFA (oleic acid) at three different concentrations (w/w), i.e., 10, 20 and 50%. Neither of the tested mWFOs interfered with microbial growth (400–600 mg L^−1^ cell-dry-weight (cdw) in all cases) as well as with the expression of the enzymes involved in PHA production (verified by SDS-PAGE, data not shown).

Further experiments were conducted using a heterogeneous set of WFOs, collected from household production (WFO- A1, A2) or from industrial activities like restaurants and other food services facilities (WFO- B1, B2), and subjected to thermal treatment or aging (Table 1). Microbial processes were carried out by supplying these WFOs to MM medium, as well as to an alternative buffered basal medium (SB), to obtain more reproducible conditions.

All the conditions reported in Table 2, except for WFO-A1, allowed biomass accumulation and over expression of the recombinant proteins (verified by SDS-PAGE, data not reported). The absence of microbial growth in WFO-A1 may be due to the presence of toxic agents generated by the heating treatment such as conjugated dienoic and trienoic acids and a wide class of peroxides^22^.

**Table 2:** Comparison of *E*. *coli* strains performances grown on different WFOs.

Strain	Medium	WFO	FFAs reduction %	cdw [mg L^−1^]
*Lipo A*	MM	A2	69	382
		B1	33	486
		B2	60	407
	**SB**	**A2**	**84**	**1**,**300**
		**B1**	**69**	**1**,**292**
		**B2**	**64**	**1**,**636**
*Lipo B*	MM	A2	40	809
		B1	58	870
		B2	44	1,157
	**SB**	**A2**	**79**	**1**,**035**
		**B1**	**56**	**1**,**115**
		**B2**	**76**	**1**,**665**

Microbial growth with different WFOs is reported as cdw, cell dry weight; FFAs reduction % was estimated by the value of unfermented oils reported in Table 1; Standard deviation of obtained data was under 15%.

Both *Lipo A* and *Lipo B* strains achieved comparable cdw in SB media supplied with WFOs (>1 g L^−1^), as well as a similar FFA reduction (Table 2). Conversely, Lipo B strain reached higher biomass accumulation with respect to Lipo A in MM, although this behaviour is not associated to a higher FFA reduction. SB conditions promoted the highest biomass accumulation in comparison to MM ones, and also resulted in the highest reduction of acid content of WFOs, which is higher than 50% in all the tested conditions. Due to the superior performances obtained, in terms of both microbial growth and FFAs reduction, PHA production was analysed for both systems in the SB conditions. Results revealed a high variability in terms of polymer yield (%PHA 0.2–10). The best results (PHA production higher than 150 mg L^−1^, corresponding to about 10% intracellular polymer percentage), were achieved from *LipoB* supplied with WFO-B2 at 72 h. At earlier growth time (24 h), polymer percentage was negligible, whilst at 48 h it was almost comparable to the value obtained at 72 h. However, prolonging the process to 72 h assured a more efficient reduction of FFA (from 50% at 48 h to ~80%). Notably, a co-polymer P(3HB-co-3HHx) with a constant molar ratio 40:60 was produced in all tested conditions by the *LipoB* system, despite the heterogeneous nature of the supplied WFOs.

**Figure 1:**
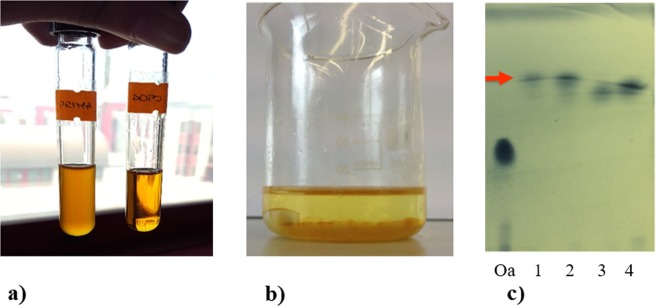
Transesterification of WFOs after microbial process. (**a**) Oils before (vial on the left) and after the bioprocess (vial on the right); (**b**) State of the final product mixtures obtained from transesterification of WFO-B1 recovered after the fermentation. Glycerol perfectly separated at the bottom of the beaker; (**c**) TLC results of product composition for transesterified oils: Oa, oleic acid; (1) WFO-B1 recovered after fermentation; (2) WFO-B2 recovered after fermentation; (3) WFO-B1 before fermentation; (4) WFO-B2 before fermentation. Red arrow indicates the band corresponding to methyl-esters.

A further step towards the validation of the proposed bioprocess consisted in the evaluation of the transesterification yield of the oils recovered at the end of the bioprocess. B1 and B2 WFOs, before and after the *LipoB* fermentation in SB medium were transesterified under commonly used basic conditions, and the reaction products analysed by TLC (Fig. 1). In fact, TLC (Fig. 1c) shows the presence of a band corresponding to fatty acid methyl esters (FAMEs)^19^, only for WFOs recovered after fermentation (lanes 1 and 2). This band was not detected for the WFOs which were not subjected to the microbial process (lanes 3 and 4).

Results obtained using *E*. *coli* strains provided a “proof of concept” of the proposed two-products bioprocess. Although the biopolymer yield is not yet satisfactory for industrial exploitation of the bioprocess, the production of a biopolymer with defined composition from complex feedstocks was achieved, leaving room for further improvement of such recombinant systems for the designed bioprocess.

### Switching to native PHA producers

*Pseudomonas resinovorans* NRRLB-2649 was selected as PHA producer because: (*i*) this strain has been characterized for its high PHAs producing abilities, from both related and non-related C-sources^16,23–26^; (*ii*) *P*. *resinovorans* is endowed with only one gene (*lip*) coding for an extracellular lipase, making the construction of a lipase free mutant easily achievable. Since the expression of lipase activity during the fermentation can hydrolyze TAGs, therefore reducing WFO value for biodiesel conversion, the proposed dual-product bioprocess was investigated comparing the performances of both *wild type* (*wt*) and the corresponding *lip* gene knocked-out mutant (*lip*^*−*^). FFAs reduction and PHA production were analyzed in cultures supplied with WFO-B2 that had undergone a long aging process (8 months, which led to further increase in its FFAs content: >10.5%). No significant difference between *lip*^*−*^ and *wt* were observed in terms of polymer production, both reaching up to 1.3 g L^−1^ PHA after 72 h, corresponding to a comparable intracellular polymer percentage (Table 3).

**Table 3:** Comparison of *P*. *resinovorans wt* and *lip*^−^ strains performances at different growth times.

	PHA [g L^−1^]		cdw [g L^−1^]		% PHA		% FFA		% Biodiesel conversion	
	*lip* ^−^	*wt*	*lip* ^−^	*wt*	*lip* ^−^	*wt*	*lip* ^−^	*wt*	*lip* ^−^	*wt*
24 h	0.15	0.16	1.66	1.51	8.9	10.6	7	8	49	3
48 h	0.58	0.52	2.49	2.63	23.4	19.8	6	7	67	65
72 h	1.33	1.35	3.80	3.92	35.1	34.5	4	5	79	72

Transesterification was carried out using 1% of NaOH. Standard deviation of reported values is under 15%.

Similar PHA production levels have been reported for *P*. *resinovorans* grown both on waste cooking oil as carbon source^27^ and on pure FFAs, *i*.*e*., coconut oil and oleic acid, in a two-stage fermentation process^23^.

Both wild type and mutant strains provide an almost comparable level of FFA reduction (Table 3). As expected by its high acid content, the un-treated WFO was not successfully transesterified, resulting in a biodiesel conversion lower than 5%. Despite the subtle difference in % FFA achieved after 24 h of fermentation by the *wt* and the mutant, a drastic worsening in biodiesel conversion was observed in the case of the *wt*. On the other hand, after 48 h of fermentation, FFAs content of recovered oil was low enough to guarantee an acceptable biodiesel conversion (yield around 70–80%) for both strains, if transesterification is performed with 1% NaOH. It is worth to note that the oil derived from *lip*^*−*^ fermentation assured the same biodiesel yield even if amount of used catalyst is reduced to 0.8%. Conversely, in the same conditions, *wt* treated oil yields a biodiesel conversion not higher than 50%. Thus, the choice of a proper strain also provides an economic and environmental benefit to the entire process, reducing costs for catalysts and wastes disposal in the biodiesel conversion step.

### Bioprocess optimization

The effect of an additional carbon source on boosting polymer production yield was investigated. Glycerol, the predominant by-product of biodiesel production, was chosen since its reuse in the proposed bioprocess, besides representing an alternative to its disposal, could close the production cycle, by turning a waste into a process feed^28^.

**Figure 2:**
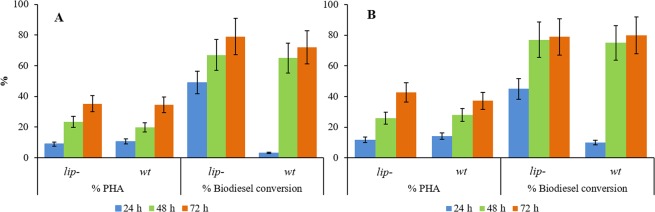
Comparison of process performances obtained by *P*. *resinovorans wt* and *lip*^−^ mutant in WFOs supplied media, in the absence (Panel A) or presence (Panel B) of 0.2% glycerol.

Two glycerol concentrations were tested: 0.2% and 0.8% v/v. When 0.2% of glycerol was added to the culture medium, PHA production at 72 h displayed about 15% increase in comparison with the corresponding condition without glycerol (Table 3 and Fig. 2). However, this increase did not get any higher when 0.8% glycerol concentration was used. On the other hand, biodiesel conversion and FFAs’ reduction were comparable to those obtained in the presence of WFO as the only carbon source (Table 4). As a fact, at the end of the fermentation, both systems are able to roughly halve oil’s FFAs content.

**Table 4:** Comparison of *P*. *resinovorans wt* and *lip*^−^ strains performances in fermentation media supplemented with 0.2% (**A**) and 0.8% glycerol (**B**). Standard deviation is under 15%.

	PHA [g L^−1^]		cdw [g L^−1^]		% PHA		% FFA		% Biodiesel conversion	
	*lip* ^−^	*wt*	*lip* ^−^	*wt*	*lip* ^−^	*wt*	*lip* ^−^	*wt*	*lip* ^−^	*wt*
**A**										
24 h	0.16	0.20	1.35	1.45	11.9	14.1	8	9	45	10
48 h	0.59	0.76	2.30	2.69	25.8	28.1	7	7	77	75
72 h	1.55	1.53	3.62	4.11	42.7	37.2	5	6	79	80
**B**										
24 h	0.18	0.13	1.22	1.46	14.7	9.2	8	9	40	10
48 h	0.83	0.68	3.11	2.81	26.8	24.2	7	7	71	75
72 h	1.50	1.23	4.22	3.97	35.5	31.1	5	6	78	77

To further push FFAs’ reduction and implement oil quality, a double-fermentation strategy was performed. After 72 h of fermentation (I step), cells were collected to analyze PHAs production while oils were recovered and used as a carbon source to feed a new culture batch (II step) for additional 72 h.

All the experiments were performed in quadruplicate. Data obtained with *lip*^−^ strain revealed a satisfactory reproducibility (standard deviation lower than 15%). At the end of the II-step, a further FFAs reduction was achieved with respect to the first 72 h (residual FFA 4%), leading to FFAs content always lower than 1%. As a fact, the latter oils could be efficiently transesterified (>80% yield) also reducing the amount of catalyst to 0.5%. Polymer production was also achieved at the end of the second step, even if both growth (2.12 g L^−1^) and PHA production levels (0.61 g L^−1^) decreased with respect to the first step (see data in Table 3 at 72 h), probably due to nutrient shortage.

On the other hand, results obtained with *wt* strain at the end of the II-step, pointed out a high variability in the FFA content of the residual oil (ranging from 3 to 15% FFAs), and consequently in biodiesel conversion (from 80 to 5%). However, the growth achieved by *wt* strain was almost comparable in all the four trials (about 5 g L^−1^ cdw), as well as the amount of PHA produced (about 2.2 g L^−1^), corresponding to about 44% PHA yield. The observed results may be due to the induction of lipase expression (triggered once most of the FFAs have been consumed), which in turn starts the consumption of TAGs and consequently increases the FFAs content. The multiplicity of factors determining lipase induction and, consequently, FFAs consumption/production for *wt* strain may explain the high variability observed.

Results indicate that the second fermentation step, although providing an improvement in PHA yield (44%) in comparison to the one step process (34.5% PHA in Table 3), is not successful in terms of FFAs reduction, if the *wt* strain is used. On the other hand, a significant FFAs reduction was achieved after the second step with the *lip*^*−*^ mutant, although nutrient shortage probably accounted for the reduced PHA production. Further studies could be focused on evaluating the role of glycerol in the two-steps process, as a solution to overcome nutrients consumption.

## Conclusions

**Figure 3:**
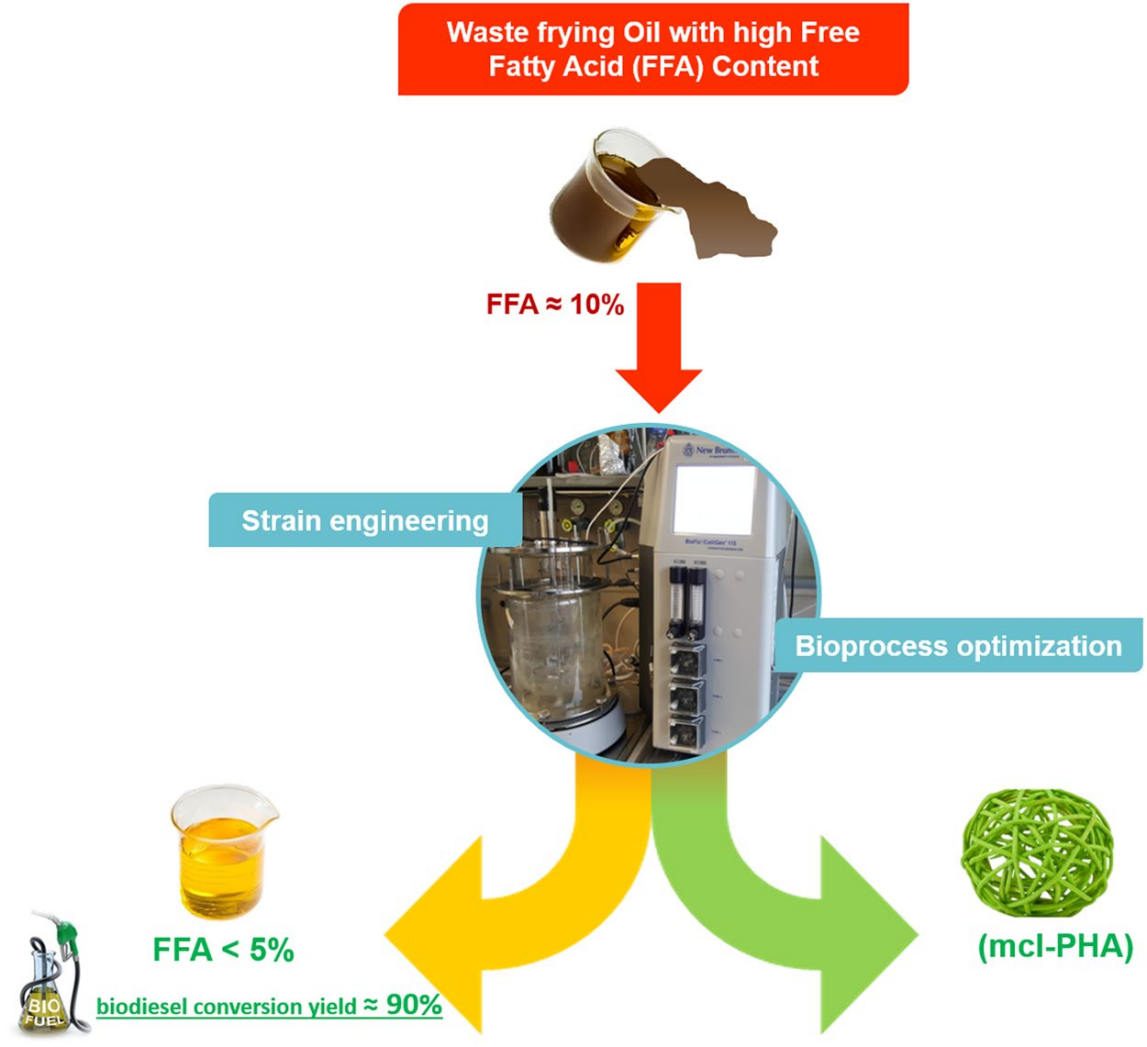
Schematic representation of the proposed multi-product process for the conversion of no/low value WFOs into biodiesel and biopolymers.

In the present work, an innovative multi-product, “biorefinery-inspired”, bioprocess for the valorisation of waste oils with high content of free fatty acids was validated on lab-scale (Fig. 3). Multiple system engineering strategies were applied to design cell factories able to simultaneously produce biopolymer from a no-low value waste and valorise the same feedstock in a secondary raw material directly convertible in biofuels.

The use of *E*. *coli* recombinant strains, although not satisfactory in terms of biopolymer yields, allowed to get a stable PHA composition, whatever was the supplied WFOs. Vice versa, polymer composition of mcl-PHA produced from *P*. *resinovorans* strains has been reported to reflect the substrates used (oleic acid, animal fats and vegetable oils) being characterized by C8 and C10 β-hydroxyacyl moieties as predominant species, and displaying different degrees of unsaturation depending on the nature of the oils^23,27^. The monomeric composition of PHAs extracted from both *wt* and *lip*^*−*^ mutant at the end of the two steps process, is consistent with literature data (C6, C12 and C14 moieties <10 mol%, C8 and C10 ~40 mol%).

A step forward towards the exploitation of the *P*. *resinovorans* based process, may derive from the control of mcl-PHA composition. The elucidation of structure/function relationships of the main actors of the PHA biosynthetic machineries, has opened the way to protein engineering approaches aimed at designing enzymes with tailored substrate specificity. This will ultimately lead to the development of engineered microorganisms producing tailor-made PHAs even from variable substrates, such as WFOs^26,29^. In this regard, the application of the process to waste streams derived from a defined source (big fast-food companies, restaurant chains), will assure a more stable PHA composition.

In conclusion, the proposed bioprocess can be considered as the “core” of a future biorefinery aimed at WFOs valorisation. To address the whole biorefinery concept, the process needs to be integrated with the crucial downstream operations, including both the extraction of the polymer from the cellular biomass, as well as the recovery of the residual culture broth and of the “treated” oil. These additional steps can be designed respecting the idea of a “zero-waste” process. For example, the residual biomass resulting after polymer extraction could be recycled by pyrolysis, producing bio-gas or energy to fulfil plant requirements. Analogously, the exhausted culture broth, could be investigated as source of biomolecules of interest (*i*.*e*. surfactants).

Taken together, the data obtained in this work laid the bases for the development of a circular economy-based bioprocess allowing PHAs production from WFOs as well as a more efficient reuse of the “purified” (de-acidified) waste oil for biodiesel production.
